# Electronic cigarettes: isolated and combined use among Brazilian adolescents in PeNSE 2019

**DOI:** 10.1590/1980-549720260024

**Published:** 2026-07-31

**Authors:** Paulo César Rodrigues Pinto Corrêa, Juliana Bottoni de Souza, Déborah Carvalho Malta

**Affiliations:** I Universidade Federal de Minas Gerais, Graduate Program in Public Health – Belo Horizonte (MG), Brazil.; II Universidade Federal de Ouro Preto, School of Medicine – Ouro Preto (MG), Brazil.; III Universidade Federal de Minas Gerais, School of Nursing, Observatory of Noncommunicable Diseases and Conditions – Belo Horizonte (MG), Brazil.; IV Universidade Federal de Minas Gerais, School of Nursing, Department of Maternal and Child Nursing and Public Health – Belo Horizonte (MG), Brazil.

**Keywords:** Adolescent, Tobacco use disorder, Nicotiana, Electronic nicotine delivery systems

## Abstract

**Objective:**

To analyze the prevalence and factors associated with e-cigarette (EC) use among adolescents aged 13-17 in Brazil, and to describe the pattern of polytobacco/nicotine product use.

**Methods:**

This is a cross-sectional study using data from 159,245 students from the 2019 National School Health Survey (PeNSE). The prevalence of EC use and combined use with other products (conventional cigarettes, hookah, and others) was estimated. The association with sociodemographic variables, mental health, and other substance use was analyzed through multivariate logistic regression, calculating adjusted Odds Ratios (aOR) and their 95% confidence intervals (95% CI).

**Results:**

The prevalence of EC use in the last 30 days was 2.82% (95%CI 2.62–3.04). Polytobacco use was significant, with dual use (EC + conventional cigarette) found in 8.56%. The combined use of all smoked products was 14.83%, and quadruple use (EC + conventional + hookah + straw) reached 14.04%. After adjustment, the factors associated with higher odds of EC use were: alcohol consumption (aOR=3.94), having friends who smoke in their presence (aOR=2.53), regular tobacco use (aOR=2.22), other drug use (aOR=2.04), feeling lonely (aOR=1.25), feeling sad (aOR=1.24), and having parents who smoke (aOR=1.17).

**Conclusions:**

E-cigarette use among Brazilian adolescents is associated with mental health factors, social influences, and the concomitant use of alcohol and other drugs. The polytobacco use is a worrying reality among these adolescents. The findings highlight the need for public policies that articulate tobacco control with mental health initiatives and strategies for preventing alcohol/other drug use.

## INTRODUCTION

In the late 1990s, Richard Doll stated that "*it is now possible to see that the medical evidence of the harm caused by smoking has been accumulating for 200 years […] the evidence was generally ignored until five case-control studies linking smoking to the development of lung cancer were published in 1950*"^1^. Thus, the harmful effects of tobacco are well established in the literature, with no safe form of use or level of exposure identified^2^.

The introduction of electronic cigarettes (ECs) has added a new dimension to the global nicotine consumption epidemic, promoting novel patterns of use among young people. These include social use in group settings and parties, as well as increased appeal driven by a wide variety of flavors, colors, and device designs, alongside their dissemination through websites, social media platforms, influencer marketing, and fake news, among other factors^3^. Two systematic reviews^4,5^, an editorial published in the *New England Journal of Medicine* ^6^, and studies focusing on specific brands^7^ have demonstrated that adolescents identify flavors as the primary factor influencing experimentation with ECs.

In Brazil, the use of these products has been increasing among adolescents, posing a significant challenge to efforts aimed at controlling the tobacco/nicotine epidemic^8^. The National Health Surveillance Agency (*Agência Nacional de Vigilância Sanitária* – Anvisa) has regulated electronic cigarettes, also referred to as electronic smoking devices (ESDs), since 2009 (Resolution No. 46/2009)^9^, and more recently through Resolution No. 855/2024^10^. The latter establishes a prohibition on the manufacture, commercialization, importation, advertising, distribution, storage, and use of these products, including accessories, parts, components, and refills intended for use with or in ESDs, encompassing both electronic cigarettes and heated tobacco products^10^. Additionally, the Resolution prohibits their use in enclosed spaces^10^. Despite these regulatory measures, such products continue to be sold in the informal market, including in small retail outlets, as well as through various online platforms and social media networks. ECs are often marketed as technological innovations or lifestyle accessories, and as part of a glamorous lifestyle, thereby attracting the attention of young people, as well as individuals who have never used conventional cigarettes.

Concerns regarding, and the reporting of, patterns of concomitant tobacco use among adolescents emerged in the early 2000s^11,12^. Polyuse is associated with increased nicotine dependence, greater difficulty in smoking cessation, and a higher incidence of tobacco-related cancers^13-17^.

The World Health Organization (WHO) Framework Convention on Tobacco Control (FCTC) represented a milestone in addressing the tobacco epidemic. To support its implementation, the WHO introduced, in 2008, the MPOWER package, comprising six of the most important and effective tobacco control measures^18^. Among these, the systematic monitoring of tobacco/nicotine use prevalence and of prevention policies is highlighted^18^. The implementation of periodic, standardized population-based surveys enables temporal comparability of data, which is essential for effective planning, impact assessment, and the refinement of tobacco control strategies.

This study aimed to describe the prevalence of ESDs use and the combined use of ESDs in the past 30 days, based on data from the most recent edition of the National School Health Survey (*Pesquisa Nacional de Saúde do Escolar* – PeNSE), and to analyze the factors associated with EC use among adolescents aged 13 to 17 years in Brazil.

## METHODS

### Study population and sample

This is a cross-sectional study based on data from the 2019 edition of PeNSE, conducted by the Brazilian Institute of Geography and Statistics (*Instituto Brasileiro de Geografia e Estatística* – IBGE) in partnership with the Ministry of Health. The survey is part of the Surveillance of Risk and Protective Factors for Chronic Noncommunicable Diseases in Brazil and addresses multiple aspects of adolescents’ lives, including health-related behaviors, care practices, risk factors, and protective factors. Previous editions of the survey were conducted in 2009, 2012, and 2015^19^.

In 2015, the survey began to include adolescents aged 13 to 17 years to ensure international comparability. In 2019, new indicators were incorporated, and the geographic scope of the sample was expanded, based on a single-stage sample of students aged 13 to 17 years enrolled in public and private schools across the following geographic levels: Brazil, Major Regions, Federative Units, capital cities, and the Federal District. Data were collected from 4,242 schools, 6,612 classes, and 159,245 students^20^. Considering enrolled students who did not respond, the non-response rate was approximately 15.4% in 2019^20^. The sample was designed to estimate population parameters for students aged 13–17 years enrolled in and attending public and private schools at the geographic levels previously described^20^. Detailed information on the sampling plan is available in the IBGE publication^20^.

### Outcome analyzed

Indicators related to the use of EC and other tobacco products were assessed in 2019, when the single question on other tobacco products was replaced by five specific questions. These questions addressed the use, in the past 30 days, of EC, hookah, clove/Bali cigarettes, and hand-rolled cigarettes (made with straw or paper), with binary response options (yes/no). Additionally, the questionnaire included the item: "IN THE LAST 30 DAYS, which of these other tobacco products did you use?", listing all previously mentioned products and including the "other" category^21^. For this question, respondents were instructed to mark all options corresponding to products they had used in the past 30 days^21^.

The prevalence of different patterns of tobacco use, including both single-product use and combined use (polyuse), was estimated.

For the outcome "e-cigarette" use in the past 30 days, explanatory variables were analyzed within the following domains: 1) sociodemographic characteristics: gender (male/female), age range (13–15 and 16–17 years), skin color (white, black, yellow, brown, or Indigenous), and cohabitation with father and/or mother (yes/no); 2) family supervision: parental or guardian awareness of the student's activities during free time in the past 30 days (yes/no) and skipping classes without authorization (yes/no); 3) mental health: feeling that no one cares about them in the past 12 months (yes/no), feeling sad (yes/no), and having close friends (one or more/none); 4) substance use: alcohol consumption in the past 30 days (yes: having consumed at least one drink or one serving of an alcoholic beverage in the 30 days prior to the survey; no: none in the 30 days prior) and drug use in the 30 days prior to the survey (yes: having used drugs in the 30 days prior to the survey; no: none in the 30 days prior); and 5) Influence of close individuals: passive smoking/people who smoke in one's presence (no and yes) and parents or guardians who smoke (no and yes).

### Statistical analysis

The descriptive analysis included the estimation of prevalences and their respective 95% confidence intervals (95%CI). Pearson's chi-square test was used to assess associations between the independent variables across groups. A p-value ≤0.05 was considered statistically significant.

The magnitude of the associations was estimated using odds ratios (OR), with their respective 95%CI, calculated for all variables of interest that presented a statistical significance level of less than 0.05 in the bivariate analysis. In the final multivariate model, all variables were included, and those with a p-value ≤0.05 were considered statistically significant.

Statistical analysis was performed using Stata software, version 14.1 (StataCorp LP, College Station, United States), employing the survey module, which incorporates post-stratification weights.

### Ethical standards

PeNSE complies with the ethical standards for research involving human subjects and was approved by the National Research Ethics Commission of the Ministry of Health (*Comissão Nacional de Ética em Pesquisa do Ministério da Saúde* – CONEP/MS), under the Certificates of Presentation for Ethical Consideration (*Certificados de Apresentação para Apreciação Ética* – CAAE) No. 1.006.48718 and No. 3.249.268.

#### Data availability statement:

The entire dataset supporting the results of this study has been/is publicly available from IBGE.

## RESULTS

**Figure 1:**
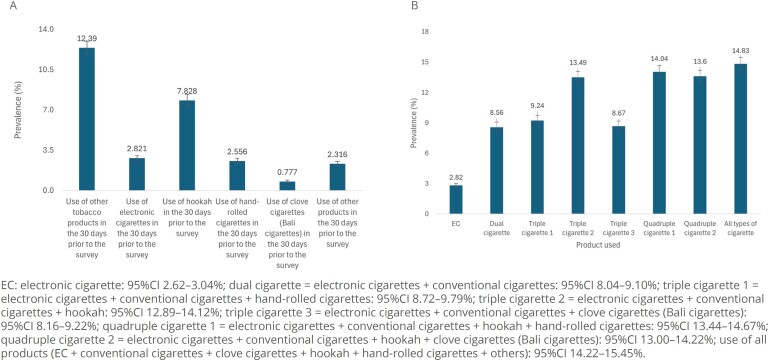
Prevalence of tobacco product use. (A) Prevalence of other tobacco products and electronic cigarettes among adolescents and (B) prevalence of combined use of conventional cigarettes and other tobacco products. National School Health Survey (*Pesquisa Nacional de Saúde do Escolhar* – PeNSE), 2019.

In 2019, the prevalence of use of other tobacco products in the past 30 days among schoolchildren aged 13 to 17 years was 12.39% (95%CI 11.85–12.95%), with the following distribution: hookah, 7.8% (95%CI 7.33–8.36%); electronic cigarettes, 2.82% (95%CI 2.62–3.04%); hand-rolled cigarettes, 2.5% (95%CI 2.31–2.83%); and Bali cigarettes, 0.8% (95%CI 0.66–0.91%) (Figure 1A). Thus, in the 2019 PeNSE, the proportion of adolescents aged 13 to 17 years reporting current use of hookah was higher than that of electronic cigarettes.

The combined use of two or more tobacco/nicotine products was verified. So-called dual use (the use of electronic and combustible cigarettes) accounted for 8.56% in the 30 days preceding the survey. Among triple-use patterns, the most frequent combination was electronic cigarettes + conventional cigarettes + hookah: 13.49%. The highest prevalence observed was the combined use of all smoked products (quintuple use plus the "others" category): 14.83%, followed by quadruple use, comprising electronic cigarettes + conventional cigarettes + hookah + straw cigarettes: 14.04% (Figure 1B).

The use of EC showed, in the 13–15-year age group, a prevalence of 2.32% (95%CI 2.09–2.58%), which was higher among those aged 16 and 17 years (3.74%; 95%CI 3.34–4.18%). The prevalence of use of these products among male individuals was 3.33% (95%CI 3.03–3.66%), whereas among females it was 2.33% (95%CI 2.09–2.59%) (Table 1).

**Table 1:** Prevalence and factors associated with electronic cigarette use among Brazilian adolescents. National School Health Survey (*Pesquisa Nacional de Saúde do Escolhar* – PeNSE) 2019.

Characteristic		%			Bivariate model			
			95%CI		OR	95%CI		p-value
			Lower	Upper		Lower	Upper	
Total		**2.82**	**2.62**	**3.04**				
Age (years)								
	13 to 15	2.32	2.09	2.58	1.00			
	16 and 17	3.74	3.34	4.18	1.63	1.39	1.91	**0.00**
Gender								
	Male	3.33	3.03	3.66	1.00			
	Female	2.33	2.09	2.59	0.69	0.60	0.79	**0.00**
Race/ethnicity								
	White	3.34	3.00	3.72	1.00			
	Black	2.79	2.33	3.35	0.83	0.66	1.04	0.11
	Yellow	3.10	2.19	4.37	0.93	0.64	1.35	0.69
	Brown	2.40	2.14	2.69	0.71	0.62	0.82	**0.00**
	Indigenous	2.44	1.71	3.49	0.73	0.49	1.07	0.10
Lives with mother and/or father								
	No	3.29	2.60	4.16	1.00			
	Yes	2.78	2.58	2.99	0.84	0.66	1.06	0.14
Parental supervision								
	No	4.03	3.60	4.51	1.00			
	Yes	2.31	2.11	2.53	0.56	0.49	0.65	**0.00**
Missed classes without authorization								
	No	2.33	2.13	2.55				
	Yes	4.83	4.32	5.39	2.12	1.86	2.42	**0.00**
Feels lonely								
	No	2.07	1.81	2.36	1.00			
	Yes	3.45	3.18	3.74	1.69	1.46	1.96	**0.00**
Sadness								
	No	2.09	1.81	2.42				
	Yes	3.20	2.95	3.47	1.55	1.31	1.82	**0.00**
Friends								
	1 or more	2.81	2.60	3.03	1.00			
	None	2.93	2.09	4.09	1.05	0.75	1.47	0.80
Regular tobacco use								
	No	1.89	1.74	2.06				
	Yes	15.68	14.30	17.16	9.65	8.53	10.92	**0.00**
Alcohol consumption								
	No	0.96	0.84	1.11	1.00			
	Yes	7.53	6.95	8.15	8.37	7.16	9.79	**0.00**
Regular drug use								
	No	2.04	1.87	2.22	1.00			
	Yes	17.08	15.26	19.06	9.90	8.54	11.48	**0.00**
Friends smoked in your presence								
	No	1.12	0.98	1.28	1.00			
	Yes	6.95	6.44	7.50	6.61	5.70	7.66	**0.00**
People smoked in your presence								
	No	2.35	2.14	2.59	1.00			
	Yes	4.06	3.60	4.58	1.76	1.50	2.06	**0.00**
Parents or guardians who smoke								
	No	2.42	2.21	2.66	1.00			
	Yes	4.08	3.65	4.56	1.71	1.49	1.98	**0.00**

The bivariate analysis (Table 1) indicated that the highest odds of e-cigarette use were observed among adolescents aged 16–17 years; those who skipped classes without permission; those who reported having no friends; those who experienced feelings of loneliness; those who used other tobacco products, alcoholic beverages, and drugs; those who had friends who smoked in their presence; and those whose parents or guardians were smokers. Living with a parent was identified as a protective factor (Table 2).

**Table 2:** Factors associated with electronic cigarette use among Brazilian adolescents, final model. National School Health Survey (*Pesquisa Nacional de Saúde do Escolhar* – PeNSE) 2019.

Characteristic		Fina Multivariate Model			
		OR*	95%CI		p-value
			Lower	Upper	
Gender					
	Male	1.00			
	Female	0.59	0.52	0.68	**0.00**
Feels lonely					
	No	1.00			
	Yes	1.25	1.05	1.49	**0.01**
Sadness					
	No	1.00			
	Yes	1.24	1.04	1.48	**0.02**
Regular tobacco use					
	No	1.00			
	Yes	2.21	1.87	2.63	**0.00**
Alcohol consumption					
	No	1.00			
	Yes	3.94	3.29	4.73	**0.00**
Regular drug use					
	No	1.00			
	Yes	2.04	1.67	2.50	**0.00**
Friends smoked in your presence					
	No	1.00			
	Yes	2.53	2.09	3.07	**0.00**
Parents or guardians who smoke					
	No	1.00			
	Yes	1.17	1.01	1.36	**0.04**

In the multivariable analysis, the following factors remained associated with a higher likelihood of EC use: feelings of loneliness (OR=1.25 95%CI 1.05–1.49); feelings of sadness (OR=1.24 95%CI 1.04–1.48); regular tobacco use (OR=2.22 95%CI 1.87–2.63); alcohol consumption (OR=3.94; 95%CI 3.29–4.73%); use of other drugs (OR=2.04; 95%CI 1.67–2.50%); having friends who smoked in their presence (OR=2.53; 95%CI 2.08–3.07%); and having parents or guardians who were smokers (OR=1.17; 95%CI 1.01–1.36%) (Table 2).

## DISCUSSION

This study assessed the association between several variables and e-cigarette use among adolescent schoolchildren in Brazil in 2019. Higher odds of use were observed among those who reported feelings of loneliness; experienced feelings of sadness; regularly used tobacco, alcohol, and other drugs; had friends who smoked in their presence; and had parents or guardians who were smokers.

This is the first Brazilian study to report polyuse, or concomitant use, of various forms of tobacco and ESDs. Polyuse of tobacco is defined as the simultaneous use of two or more tobacco products^22-36^. Among the different patterns of polyuse, dual use (conventional and electronic cigarettes) was the least common, at 8.56%, whereas the combined use of all smoked products was the most prevalent, at 14.83%.

PeNSE reported a prevalence of current use of conventional cigarettes of 6.8%^20^, whereas the present study found that the use of other tobacco products in the 30 days preceding PeNSE 2019 was 12.39%. In a systematic review and meta-analysis conducted by Barufaldi et al.^22^, 22 longitudinal studies published between 2016 and 2020 from different countries were identified, totaling 97,659 participants for the outcome of experimentation. The use of EC increased the risk of experimentation with regular (or conventional) cigarettes by nearly three and a half times (relative risk — RR=3.42; 95%CI 2.81–4.15) and increased the risk of current smoking by more than fourfold (RR=4.32; 95%CI 3.13–5.94)^22^.

Among the analyses of factors associated with EC use, the highest odds of use were observed among individuals who reported feelings of loneliness and experienced feelings of sadness. Tobacco use has a well-established bidirectional relationship with mental health, whereby mental health symptoms predict subsequent smoking, and smoking contributes to the worsening of mental health symptoms^23,24^. VanFrank et al.^25^ analyzed self-reported data from the National Youth Tobacco Survey 2024 to examine EC use and symptoms of depression and anxiety among American middle school (grades 6–8) and high school (grades 9–12) students. Among students who had previously used a tobacco product, 42.9% reported current use, while 43.6% of those who had previously used EC reported current ESD use. In 2024, 42.1% of young people who currently used e-cigarettes reported moderate to severe symptoms of depression and anxiety, compared with 21.0% of those who did not use these products^25^. Among young people who used e-cigarettes, those with moderate to severe symptoms of depression and anxiety (*vs*. mild symptoms) more frequently reported symptoms of nicotine dependence^25^. These findings suggest that, similarly to what has been established for conventional cigarettes, a bidirectional relationship may exist between EC use and mental health conditions. However, due to the cross-sectional design, it is not possible to determine whether e-cigarette use preceded the worsening of mental health symptoms, whether worsening mental health symptoms preceded e-cigarette use, or whether both directions are present. This temporal relationship can only be adequately assessed through longitudinal cohort studies; therefore, the associations identified in this study should be interpreted with caution.

The higher likelihood of EC use among adolescents who had friends who smoked in their presence and whose parents or guardians were smokers is consistent with findings reported in the literature. Le^26^ also identified family and friends as important factors in the initiation of ESD use among adolescents. Bailey et al.^27^ reported that parental ESD use predicts e-cigarette use among adolescent and young adult offspring, beyond the effect of parental cigarette use. According to Groom et al.^28^, peer and friend influence constitutes the primary social driver and reinforcer of ESD use.

The promotion and advertising of electronic cigarettes and other tobacco and nicotine products constitute major drivers of their use^29^. Peer influence was examined by Kim et al.^30^, who demonstrated that marketing EC-related lifestyles to individuals of the same age group or to peers is a prominent feature of ESD advertising targeting young people on social media platforms. Exposure to EC advertising has been associated with experimentation among adolescents and young adults^31^. Abreu et al.^32^ recently published the first investigation of tobacco and nicotine product advertising on social media platforms in Brazil, reporting a high prevalence of content directed at younger audiences, consistent with findings from international studies. PeNSE 2019, as in previous editions, did not include data on exposure of Brazilian adolescents to the promotion and advertising of tobacco/nicotine products on social media platforms.

This investigation identified a higher prevalence of EC use among adolescents who used other psychoactive substances and consumed alcohol. Analysis of comparable indicators among 9th-grade students in PeNSE, conducted in Brazilian capital cities from 2009 to 2019, demonstrated that, despite the legal prohibition of alcohol consumption among adolescents, 67.9% of students had experimented with alcoholic beverages and 25.5% had consumed them in the 30 days preceding the survey^33^. The literature indicates that early initiation of EC use (defined as use in the ninth grade or earlier) is significantly associated with an increased likelihood of cigarette smoking and engagement in other substance use behaviors^22,34^. E-cigarette use is generally preceded by alcohol consumption, cigarette smoking, and marijuana use^34^. Notably, Milicic and Leatherdale^35^ demonstrated that adolescents who engage in heavy alcohol consumption or marijuana use have a higher likelihood of e-cigarette use compared with cigarette smokers. Audrain-McGovern et al.^36^ reported that EC and hookah use at age 14 are associated with a 3.6- to 4-fold increase in the OR of initiating and currently using marijuana two years later. Furthermore, early adolescent use of EC and hookah more than doubled the OR of concurrent tobacco and marijuana use during mid-adolescence.

Bertoni and Szklo^37^ reported data from the 2019 Surveillance of Risk and Protective Factors for Chronic Diseases by Telephone Survey (*Vigilância de Fatores de Risco e Proteção para Doenças Crônicas por Inquérito Telefônico* – Vigitel), which interviewed 52,443 individuals aged 18 years old or older from the 26 Brazilian state capitals and the Federal District. There was a higher prevalence of current use of ESD among individuals who reported abusive alcohol consumption compared with those who did not drink in this manner (6.33 *vs*. 1.40%)^34^. This difference was more pronounced in the 18–24-year age group, an age stratum immediately above that evaluated by PeNSE^37^.

These data clearly indicate the need for prevention programs that are not limited solely to the domain of tobacco/nicotine control. The theoretical models of school-based tobacco prevention programs are quite diverse, including information-based curricula, social influence approaches, and combined multimodal social skills strategies^38^. The Education Against Tobacco (EAT) program is an intervention whose effectiveness has already been demonstrated in Brazil^39,40^.

However, effective public policies for drug use prevention and their articulation with tobacco/nicotine control remain insufficient. In this context, Resolution No. 08 of the National Council on Drug Policies (*Conselho Nacional de Políticas sobre Drogas* – CONAD) establishes the approval of the National Drug Policy Plan 2022–2027^41^. The Plan is essentially limited to "*creating a platform for the identification and subsequent evaluation of the various initiatives carried out in Brazil on prevention*"^41^. In other words, the sole guideline until 2027 is to catalog actions for their subsequent evaluation.

The official publication of PeNSE 2019 investigated how respondents who had already tried cigarettes obtained tobacco products: 37.5% of experimenters reported acquiring them in commercial establishments, such as stores, bars, bakeries, or newsstands^20^. Cabral et al.^42^ published a scoping review and a technical report demonstrating the importance of restricting points of sale as a measure to prevent smoking^43^. They emphasize that, in addition to sales to minors being illegal, the sale of single cigarettes (individual cigarettes rather than packs) still occurs. They also highlight that the multiplicity of points of sale (POS) facilitates access to tobacco products, particularly given their presence near educational institutions^42,43^. Low-income neighborhoods generally present a higher density of tobacco POS and higher prevalence of tobacco use, contributing to health inequities^42,43^.

A bill is currently under consideration in the Federal Senate of Brazil that would prohibit the sale of "smoking products" in primary and secondary schools, health services, places where food is sold or consumed, supermarkets, convenience stores, and newsstands^44^. If approved, it would represent a major advancement in tobacco/nicotine control policies in the country. Greater enforcement of the ban on the sale of ESD is also necessary. Perez et al. identified widespread illegal sales of ESD and other tobacco products on websites, social media, and e-commerce platforms, in defiance of the restrictions established by Brazilian legislation^45^.

Among the study's limitations, the attribution of causality between the indicators and the investigated outcomes stands out, due to the cross-sectional design, recall bias, and potential misinterpretation of questions in the self-administered questionnaire. There is also selection bias, as the sample is restricted to enrolled students, excluding out-of-school adolescents, a potentially more vulnerable group. However, PeNSE employs a robust methodology that encompasses all major regions, Federative Units, and capital cities, thereby approximating the Brazilian reality.

The study highlighted the association between EC use and mental distress (loneliness and sadness), male gender, and the influence of parents or peers who use these products. Understanding these factors is essential to addressing the increasing popularity of nicotine use among young people. Notably, the findings also underscore the concerning pattern of polytobacco use and its strong correlation with other substances: individuals who use alcohol and illicit drugs were more likely to use electronic cigarettes.

Therefore, efforts to prevent the use of EC should not be limited solely to the domain of tobacco/nicotine control. It is urgent to coordinate tobacco, alcohol, and drug control policies through integrated actions orchestrated by the National Anti-Drug Secretariat. Comprehensive strategies are required, including price and tax increases; monitoring of the illegal sale of loose cigarettes and EC; restrictions on tobacco product sales locations; media campaigns (including social media); and the provision of smoking cessation medications for specific groups of young people within the Unified Health System (*Sistema Único de Saúde* – SUS). Such measures are essential to reduce the initiation and prevalence of tobacco and nicotine use among young Brazilians.

## References

[1] Doll R (1998). Uncovering the effects of smoking: historical perspective. Stat Methods Med Res.

[2] World Health Organization & WHO Tobacco Free Initiative (2006). Tobacco: deadly in any form or disguise [Internet].

[3] World Health Organization (WHO) (2025). WHO report on the global tobacco epidemic, 2025: warning about the dangers of tobacco.

[4] Livingstone-Banks J, Travis N, Conde M, Chen Y (Crystal), Zi P, Jarman H (2025). The impacts of e-cigarette flavours: An overview of systematic reviews. Addiction.

[5] Zare S, Nemati M, Zheng Y (2018). A systematic review of consumer preference for e-cigarette attributes: Flavor, nicotine strength, and type. PLoS One.

[6] Drazen JM, Morrissey S, Campion EW (2019). The dangerous flavors of e-cigarettes. N Engl J Med.

[7] Kong G, Bold KW, Morean ME, Bhatti H, Camenga DR, Jackson A Appeal of JUUL among adolescents. Drug Alcohol Depend 2019.

[8] Malta DC, Morais ÉAH, Silva AG, Souza JB, Gomes CS, Santos FM (2024). Mudanças no uso do tabaco entre adolescentes brasileiros e fatores associados: Pesquisa Nacional de Saúde do Escolar. Ciên Saúde Coletiva.

[9] Agência Nacional de Vigilância Sanitária (Anvisa) (2009). Resolução de Diretoria Colegiada da Anvisa: RDC nº 46. Proíbe a comercialização, a importação e a propaganda de quaisquer dispositivos eletrônicos para fumar, conhecidos como cigarro eletrônico. Diário Oficial da União [Internet].

[10] Agência Nacional de Vigilância Sanitária (Anvisa) (2024). Resolução da Diretoria Colegiada RDC nº 855, de 23 de abril de 2024 [Internet].

[11] Wetter DW, McClure JB, Moor C, Cofta-Gunn L, Cummings S, Cinciripini PM (2002). Concomitant use of cigarettes and smokeless tobacco: prevalence, correlates, and predictors of tobacco cessation. Prev Med.

[12] Gilpin EA, Pierce JP (2003). Concurrent use of tobacco products by California adolescents. Prev Med.

[13] Bombard JM, Pederson LL, Nelson DE, Malarcher AM (2007). Are smokers only using cigarettes? Exploring current polytobacco use among an adult population. Addict Behav.

[14] Centers for Disease Control and Prevention (CDC) (2010). Any tobacco use in 13 States --- behavioral risk factor surveillance system, 2008. MMWR Morb Mortal Wkly Rep [Internet].

[15] Centers for Disease Control and Prevention (CDC) (2010). Tobacco use among middle and high school students --- United States, 2000-2009. MMWR Morb Mortal Wkly Rep [Internet].

[16] Fagan P, Moolchan ET, Lawrence D, Fernander A, Ponder PK Identifying health disparities across the tobacco continuum. Addiction 2007.

[17] King BA, Dube SR, Tynan MA (2012). Current tobacco use among adults in the United States: findings from the national adult tobacco survey. Am J Public Health.

[18] World Health Organization (2008). Report on the Global Tobacco Epidemic, 2008: The MPOWER Package [Internet].

[19] Oliveira MM, Campos MO, Andreazzi MAR, Malta DC, Oliveira MM, Campos MO (2017). Características da Pesquisa Nacional de Saúde do Escolar - PeNSE. Epidemiol Serv Saúde.

[20] IBGE (2021). Pesquisa Nacional de Saúde do Escolar PENSE 2019 [Internet].

[21] IBGE (2019). Pesquisa Nacional de Saúde do Escolar 2019: questionário do aluno [Internet].

[22] Barufaldi LA, Guerra RL, Albuquerque RCR, Nascimento A, Chança RD, Souza MC (2021). Risco de iniciação ao tabagismo com o uso de cigarros eletrônicos: revisão sistemática e meta-análise. Ciên Saúde Coletiva.

[23] Farooqui M, Shoaib S, Afaq H, Quadri S, Zaina F, Baig A (2023). Bidirectionality of smoking and depression in adolescents: a systematic review. Trends Psychiatry Psychother.

[24] Park S, Romer D (2007). Associations between smoking and depression in adolescence: an integrative review. J Korean Acad Nurs.

[25] VanFrank B, Williams TR, Alcantara IC, Hertz M, Al-Shawaf M, Meyers C (2025). E-cigarette use and symptoms of depression and anxiety among US middle and high school students. Prev Chronic Dis.

[26] Le TTT (2023). Key risk factors associated with electronic nicotine delivery systems use among adolescents. JAMA Netw Open.

[27] Bailey JA, Epstein M, Kosterman R (2022). Parent ENDS use predicts adolescent and young adult offspring ENDS use above and beyond parent cigarette use. Addict Behav.

[28] Groom AL, Vu THT, Landry RL, Kesh A, Hart JL, Walker KL (2021). The influence of friends on teen vaping: a mixed-methods approach. Int J Environ Res Public Health.

[29] U.S. Department of Health and Human Services (2012). Cigarette smoking among young people in the United States. Preventing tobacco use among youth and young adults: a report of the surgeon general [Internet].

[30] Kim M, Olson S, Jordan JW, Ling PM (2020). Peer crowd-based targeting in E-cigarette advertisements: a qualitative study to inform counter-marketing. BMC Public Health.

[31] Collins L, Glasser AM, Abudayyeh H, Pearson JL, Villanti AC (2019). E-cigarette marketing and communication: how e-cigarette companies market e-cigarettes and the public engages with e-cigarette information. Nicotine Tob Res.

[32] Abreu BM, Assimos RDN, Ricardino LM, Frazão JDS, Piras SS, Castello Branco PA (2025). The profile of illegal advertising of tobacco and nicotine products on social networks in Brazil. Tob Prev Cessat.

[33] IBGE (2019). Coordenação de População e Indicadores Sociais. Pesquisa nacional de saúde do escolar: análise de indicadores comparáveis dos escolares do 9o ano do ensino fundamental - municípios das capitais - 2009/2019 [Internet].

[34] McCabe SE, West BT, McCabe VV (2018). Associations between early onset of e-cigarette use and cigarette smoking and other substance use among US adolescents: a national study. Nicotine Tob Res.

[35] Milicic S, Leatherdale ST (2017). The associations between e-cigarettes and binge drinking, marijuana use, and energy drinks mixed with alcohol. J Adolesc Heal.

[36] Audrain-McGovern J, Stone MD, Barrington-Trimis J, Unger JB, Leventhal AM (2018). Adolescent e-cigarette, hookah, and conventional cigarette use and subsequent marijuana use. Pediatrics.

[37] Bertoni N, Szklo AS (2021). Dispositivos eletrônicos para fumar nas capitais brasileiras: prevalência, perfil de uso e implicações para a Política Nacional de Controle do Tabaco. Cad Saúde Pública.

[38] Corrêa PC (2021). Sociedade Brasileira de Pneumologia e Tisiologia (ed.). Tabagismo: prevenção e tratamento.

[39] Xavier LEDF, Bernardes-Souza B, Lisboa OC, Seeger W, Groneberg DA, Tran TA (2017). A medical student–delivered smoking prevention program, education against tobacco, for secondary schools in Brazil: study protocol for a randomized trial. JMIR Res Protoc.

[40] Lisboa OC, Bernardes-Souza B, Xavier LEF, Almeida MR, Corrêa PCRP, Brinker TJ (2019). A smoking prevention program delivered by medical students to secondary schools in Brazil called "Education against Tobacco": Randomized controlled trial. J Med Internet Res.

[41] Brasil. Ministério da Justiça e Segurança Pública (2022). Conselho Nacional de Políticas sobre Drogas. Resolução CONAD nº 08, de 27 de setembro de 2022.

[42] Cabral LMS, Giongo MJDS, Jardim FN, Carvalho AM (2023). Restrição da venda de produtos de tabaco apenas em tabacarias: uma medida necessária para o fortalecimento da Política Nacional de Controle do Tabaco. Physis.

[43] Instituto Nacional do Câncer (2023). Experiências e cenários existentes sobre a restrição da venda de produtos de tabaco apenas em tabacarias.

[44] Brasil (2023). Projeto de Lei nº 4605, de 2023 - Altera a Lei nº 9.294, de 15 de julho de 1996, para proibir a venda de produtos de tabaco nos locais que especifica.

[45] Perez CA, Veloso S, Viegas JR (2024). Venda ilegal de produtos de tabaco e dispositivos eletrônicos para fumar (DEF) na internet.

