# Supplementary material for: Electronic cigarettes: isolated and combined use among Brazilian adolescents in PeNSE 2019
Source: Rev Bras Epidemiol. 2026 Jul 31;29:e260024. doi: 10.1590/1980-549720260024 (PMC13426523; doi:10.1590/1980-549720260024)
Supplement: Translation

# Translation: Cigarros eletrônicos: uso isolado e combinado entre jovens brasileiros na Pesquisa Nacional de Saúde do Escolar 2019 — uma preocupante realidade

**DOI:** 10.1590/1980-549720260024.2

**Authors:** Paulo César Rodrigues Pinto Corrêa, Juliana Bottoni de Souza, Déborah Carvalho Malta

**Affiliations:** I Universidade Federal de Minas Gerais, Programa de Pós-Graduação em Saúde Pública – Belo Horizonte (MG), Brasil.; II Universidade Federal de Ouro Preto, Escola de Medicina – Ouro Preto (MG), Brasil.; III Universidade Federal de Minas Gerais, Escola de Enfermagem, Observatório de Doenças e Agravos Não Transmissíveis – Belo Horizonte (MG), Brasil.; IV Universidade Federal de Minas Gerais, Escola de Enfermagem, Departamento de Enfermagem Materno-Infantil e Saúde Pública – Belo Horizonte (MG), Brasil.

**Keywords:** Adolescente, Tabagismo, Nicotiana, Sistemas eletrônicos de liberação de nicotina

## Abstract

**Objetivo:**

Analisar a prevalência e os fatores associados ao uso de cigarros eletrônicos (CE) entre adolescentes de 13 a 17 anos no Brasil, bem como descrever o padrão de poliuso de produtos de tabaco/nicotina.

**Métodos:**

Estudo transversal com dados de 159.245 escolares da Pesquisa Nacional de Saúde do Escolar (PeNSE) de 2019. Foram estimadas prevalências de uso de CE e uso combinado de produtos. A associação com variáveis sociodemográficas, de saúde mental e uso de outras substâncias foi analisada por regressão logística multivariada, com cálculo de *odds ratios* (OR) ajustados e seus intervalos de confiança de 95% (IC95%).

**Resultados:**

A prevalência de uso atual de CE foi 2,82% (IC95% 2,62–3,04), e de uso dual 8,56%. A utilização combinada de todos os produtos fumados foi 14,83%, enquanto o uso quádruplo (CE + convencional + narguilé + palha) atingiu 14,04%. Após ajuste, os fatores com maior chance de uso de CE foram: consumo de bebidas alcoólicas (OR=3,94), ter amigos que fumam na sua presença (OR=2,53), uso regular de tabaco (OR=2,22), uso de outras drogas (OR=2,04), sentir-se solitário (OR=1,25), sentir-se triste (OR=1,24) e ter pais fumantes (OR=1,17).

**Conclusão:**

O uso de CE entre adolescentes brasileiros está associado a fatores de saúde mental, influências sociais e ao uso concomitante de álcool e outras drogas. O poliuso de produtos de nicotina é uma realidade preocupante. Estes achados reforçam a necessidade de políticas públicas que articulem o controle do tabaco com ações de saúde mental e de prevenção ao uso de álcool e outras drogas.

## INTRODUÇÃO

No final dos anos 1990, Richard Doll escreveu "*agora é possível ver que as evidências médicas dos danos causados pelo fumo vêm se acumulando há 200 anos […] as evidências foram geralmente ignoradas até que cinco estudos de caso-controle relacionando o tabagismo ao desenvolvimento de câncer de pulmão foram publicados em 1950*"^1^. Assim, as evidências dos malefícios do tabaco estão claramente definidas na literatura, não existindo nem uma forma de uso nem nível seguro de exposição ao tabaco^2^.

A introdução dos cigarros eletrônicos (CE) tem trazido uma nova faceta da epidemia global de consumo de nicotina, introduzindo novas formas de uso entre jovens, seja pelo uso coletivo, em grupos e festas; seja pelo apelo em função de opções de sabores, cores, formatos, pela sua popularização de venda nos *sites* e redes sociais, *marketing* por *influencers*, "*fake news*" etc.^3^. Duas revisões sistemáticas^4,5^, um editorial na revista de prestígio *New England Journal of Medicine* ^6^ e estudos específicos de determinada marca^7^ mostraram que adolescentes consideram os sabores o fator mais importante para a experimentação dos CE.

No Brasil, a utilização desses produtos tem aumentando entre adolescentes, criando um grande desafio no enfrentamento da epidemia do tabaco/nicotina^8^. A Agência Nacional de Vigilância Sanitária (Anvisa) regula os cigarros eletrônicos ou dispositivos eletrônicos para fumar (DEF) desde 2009 (Resolução n.º 46/2009)^9^ e mais recentemente pela Resolução n.º 855/2024^10^. Esta última determina que estão proibidos fabricação, comercialização, importação, propaganda, distribuição, armazenamento e importação desses produtos, incluindo acessórios, peças, partes e refis destinados ao uso com/em DEF, que incluem os cigarros eletrônicos e os de tabaco aquecido^10^. Adicionalmente, a Resolução proíbe que eles sejam consumidos em ambientes fechados^10^. Entretanto, eles são vendidos pelo mercado informal em comércios populares, por plataformas *online* variadas e redes sociais. Os CE são apresentados como novidade tecnológica ou *gadget* e/ou parte de um estilo de vida glamouroso, chamando a atenção dos jovens e também de pessoas que nunca utilizaram cigarros convencionais.

A preocupação e o relato na literatura de formas de uso concomitante de tabaco por adolescentes começou a ser feita já no início dos anos 2000^11,12^. O poliuso está associado a maior dependência de nicotina, maior dificuldade para parar de fumar e maior incidência de cânceres relacionados ao tabagismo^13-17^.

A Convenção-Quadro de Controle do Tabaco (CQCT) da Organização Mundial da Saúde (OMS) foi o primeiro passo na luta contra a pandemia. Para a efetivação da CQCT, a OMS estabeleceu, em 2008, o pacote MPOWER, um conjunto das seis políticas mais importantes e eficazes de controle do tabaco^18^. Monitorar cuidadosamente a prevalência de uso de tabaco/nicotina e as políticas de prevenção é uma dessas seis políticas^18^. A realização de pesquisas populacionais periódicas e padronizadas permite a comparabilidade temporal dos dados, essencial para o planejamento eficiente, a mensuração de impacto e o ajuste das estratégias de controle do tabagismo.

O objetivo deste estudo é descrever as prevalências de uso de DEF e de usos combinados de produtos nos últimos 30 dias na última edição da Pesquisa Nacional de Saúde do Escolar (PeNSE) e analisar os fatores associados ao uso do CE entre os jovens de 13 a 17 anos no Brasil.

## MÉTODOS

### População estudada e amostra

Estudo transversal com dados da edição de 2019 da PeNSE, realizada pelo Instituto Brasileiro de Geografia e Estatística (IBGE) em parceria com o Ministério da Saúde. A Pesquisa compõe a Vigilância dos Fatores de Risco e Proteção das Doenças Crônicas não Trasmissíveis do Brasil, abordando diversos aspectos da vida dos adolescentes, como hábitos, cuidados, fatores de risco e proteção para a sua saúde. As edições anteriores da pesquisa ocorreram em 2009, 2012 e 2015^19^.

Em 2015, o inquérito começou a incluir jovens de 13 a 17 anos, a fim de garantir comparabilidade internacional. Em 2019 houve a incorporação de novos indicadores e a ampliação da abrangência geográfica da amostra, sendo utilizada uma única amostra de estudantes de 13 a 17 anos de idade, de escolas públicas e privadas, para os seguintes níveis geográficos: Brasil, Grandes Regiões, Unidades da Federação, municípios das capitais e Distrito Federal. Coletaram-se dados em 4.242 escolas, 6.612 turmas e entre 159.245 escolares^20^). Considerando-se os alunos matriculados e não respondentes, a perda amostral foi de aproximadamente de 15,4% em 2019^20^. A amostra foi dimensionada para estimar parâmetros populacionais para os estudantes de 13–17 anos matriculados e frequentando as escolas públicas e privadas, para os níveis geográficos já citados^20^. Detalhes do plano amostral constam da publicação do IBGE^20^.

### Desfecho analisado

Foram avaliados os indicadores referentes ao uso de CE e de outros produtos de tabaco em 2019, ano em que a pergunta única sobre outros produtos do tabaco foi descontinuada e desmembrada em cinco perguntas, que indagavam sobre o uso nos últimos 30 dias de CE, narguilé, cigarros de cravo/Bali e cigarros enrolados à mão (palha ou papel), com opções de resposta binárias (sim/não). Além disso, havia a pergunta "NOS ÚLTIMOS 30 DIAS, qual/is desse(s) outros produtos do tabaco você usou", e listavam-se todos os produtos já mencionados e incluía-se a categoria "outros"^21^. Para esta última pergunta o respondente marcava com um X todas as opções que utilizara nos últimos 30 dias^21^.

Foi calculada a prevalência dos diferentes usos do tabaco, de forma única e combinada (poliuso).

Para o desfecho cigarro eletrônico "(*e-cigarette*)" nos últimos 30 dias, foram analisadas as variáveis explicativas nos seguintes contextos: 1) sociodemográficas: sexo (masculino/feminino), faixa etária (13–15, 16–17 anos), cor da pele (branca, preta, amarela, parda ou indígena), morar com pai e ou mãe (não e sim); 2) supervisão familiar: conhecimento dos pais ou responsáveis sobre realmente o que o escolar estava fazendo em seu tempo livre nos últimos 30 dias (não e sim), faltar às aulas sem autorização (sim e não); 3) saúde mental: sentir que ninguém se preocupa com você durante os últimos 12 meses (não e sim), sentir-se triste (não e sim), ter amigos próximos (um ou mais e não); 4) uso de substâncias: uso de bebidas alcoólicas nos últimos 30 dias (sim: ter tomado pelo menos um copo ou uma dose de bebida alcoólica nos 30 dias anteriores à pesquisa e não: nenhum dos 30 dias anteriores) e uso de drogas nos 30 dias anteriores à pesquisa (sim: ter usado droga nos 30 dias anteriores a pesquisa e não: nenhum dos 30 dias anteriores); 5) influência de pessoas próximas: fumante passivo/pessoas que fumam na sua presença (não e sim) e pais ou responsáveis fumantes (não e sim).

### Análise estatística

A análise descritiva incluiu o cálculo das prevalências e os respectivos intervalos de confiança de 95% (IC95%). Foi usado o teste do qui-quadrado de Pearson para verificar associação entre as variáveis independentes segundo os grupos. Foi considerado como significância estatística o valor p≤0,05.

A magnitude das associações foi estimada pela *odds ratio* (OR), com os respectivos IC95%, e elas foram realizadas para todas as variáveis de interesse relacionadas com nível de significância estatística inferior a 0,05 na análise bivariada. No modelo multivariado final, foram inseridas todas as variáveis e consideraram-se significativas as variáveis com valor de p menor ou igual a 0,05.

A análise estatística foi feita no *software* Stata, versão 14.1 (StataCorp LP, College Station, Estados Unidos), empregando-se o módulo *survey*, que considera os pesos de pós-estratificação.

### Normas éticas

A PeNSE cumpre as normas de pesquisa com seres humanos, tendo sido aprovada pela Comissão Nacional de Ética em Pesquisa do Ministério da Saúde (CONEP/MS), sob os Certificados de Apresentação para Apreciação Ética (CAAE) nº 1.006.48718 e nº 3.249.268.

#### Declaração de disponibilidade de dados:

Todo o conjunto de dados que sustenta os resultados deste estudo foi/está disponível publicamente no IBGE.

## RESULTADOS

Em 2019 a prevalência de uso de outros produtos de tabaco nos últimos 30 dias entre escolares de 13 a 17 anos foi de 12,39% (IC95% 11,85–12,95%), variando de narguilé 7,8% (IC95% 7,33–8,36%); cigarros eletrônicos 2,82% (IC95% 2,62–3,04%); cigarro de palha 2,5% (IC95% 2,31–2,83%); cigarro de Bali 0,8% (IC95% 0,66–0,91%) (Figura 1A). Assim, na PeNSE 2019 os jovens de 13 a 17 anos faziam uso atual de narguilé em proporção maior do que a de cigarros eletrônicos. Verificamos a utilização combinada de dois ou mais produtos de tabaco/ nicotina. O chamado uso dual (uso de cigarro eletrônico e de cigarro comburente) foi de 8,56% nos 30 dias anteriores à pesquisa. Entre as formas de uso triplo houve maior utilização da combinação de cigarros eletrônicos + convencional + narguilé : 13,49%. A maior frequência encontrada foi a utilização combinada de todos os produtos fumados (uso quintúplo mais a categoria outros): 14,83%, seguida pelo uso quádruplo, na combinação de cigarros eletrônicos + convencional + narguilé + palha: 14,04% (Figura 1B).

**Figura 1:** Prevalências de uso de produtos de tabaco. (A) Prevalência de outros produtos de tabaco e cigarros eletrônicos entre adolescentes e (B) prevalências de uso combinado do cigarro convencional e outros produtos do tabaco Pesquisa Nacional de Saúde do Escolhar (PeNSE), 2019.

O uso de CE mostrou, no grupo de 13 a 15 anos, prevalência de 2,32% (IC95% 2,09–2,58%), tendo sido mais elevada entre aqueles com 16 e 17 anos (3,74%; IC95% 3,34–4,18%). A prevalência de uso dos mesmos produtos entre os indivíduos do sexo masculino foi de 3,33% (IC95% 3,03–3,66%), enquanto no sexo feminino foi de 2,33% (IC95% 2,09–2,59%) (Tabela 1).

**Tabela 1:** Prevalência e fatores associados ao uso de cigarro eletrônico entre os adolescentes brasileiros. Pesquisa Nacional de Saúde do Escolhar (PeNSE) 2019.

Variável		%			Modelo bivariado			
			IC95%		OR	IC95%		Valor p
			Inferior	Superior		Inferior	Superior	
Total		**2,82**	**2,62**	**3,04**				
Idade (anos)								
	13 a 15	2,32	2,09	2,58	1,00			
	16 e 17	3,74	3,34	4,18	1,63	1,39	1,91	**0,00**
Sexo								
	Masculino	3,33	3,03	3,66	1,00			
	Feminino	2,33	2,09	2,59	0,69	0,60	0,79	**0,00**
Raça								
	Branca	3,34	3,00	3,72	1,00			
	Preta	2,79	2,33	3,35	0,83	0,66	1,04	0,11
	Amarela	3,10	2,19	4,37	0,93	0,64	1,35	0,69
	Parda	2,40	2,14	2,69	0,71	0,62	0,82	**0,00**
	Indígena	2,44	1,71	3,49	0,73	0,49	1,07	0,10
Mora com mãe e ou pai								
	Não	3,29	2,60	4,16	1,00			
	Sim	2,78	2,58	2,99	0,84	0,66	1,06	0,14
Supervisão familiar								
	Não	4,03	3,60	4,51	1,00			
	Sim	2,31	2,11	2,53	0,56	0,49	0,65	**0,00**
Faltar às aulas sem autorização								
	Não	2,33	2,13	2,55				
	Sim	4,83	4,32	5,39	2,12	1,86	2,42	**0,00**
Sentir-se solitário								
	Não	2,07	1,81	2,36	1,00			
	Sim	3,45	3,18	3,74	1,69	1,46	1,96	**0,00**
Tristeza								
	Não	2,09	1,81	2,42				
	Sim	3,20	2,95	3,47	1,55	1,31	1,82	**0,00**
Amigos								
	1 ou mais	2,81	2,60	3,03	1,00			
	Não tenho	2,93	2,09	4,09	1,05	0,75	1,47	0,80
Tabaco regular								
	Não	1,89	1,74	2,06				
	Sim	15,68	14,30	17,16	9,65	8,53	10,92	**0,00**
Bebidas alcóolicas								
	Não	0,96	0,84	1,11	1,00			
	Sim	7,53	6,95	8,15	8,37	7,16	9,79	**0,00**
Drogas regularmente								
	Não	2,04	1,87	2,22	1,00			
	Sim	17,08	15,26	19,06	9,90	8,54	11,48	**0,00**
Amigos fumaram na sua presença								
	Não	1,12	0,98	1,28	1,00			
	Sim	6,95	6,44	7,50	6,61	5,70	7,66	**0,00**
Pessoas que fumaram na sua presença								
	Não	2,35	2,14	2,59	1,00			
	Sim	4,06	3,60	4,58	1,76	1,50	2,06	**0,00**
Pais ou responsáveis fumantes								
	Não	2,42	2,21	2,66	1,00			
	Sim	4,08	3,65	4,56	1,71	1,49	1,98	**0,00**

A análise bivariada (Tabela 1) mostrou que as maiores chances de usar cigarro eletrônico foram entre os adolescentes de 16 a 17 anos; que faltaram as aulas sem autorização; que relataram não ter amigos; que se sentem solitários, que faziam uso de outros produtos do tabaco, de bebidas alcóolicas e drogas; que tinham amigos que fumaram na sua presença e cujos pais ou responsáveis eram fumantes. Constituiu fator de proteção morar com pai e/ou mãe (Tabela 2).

**Tabela 2:** Fatores associados ao uso de cigarro eletrônico entre os adolescentes brasileiros, modelo final. Pesquisa Nacional de Saúde do Escolhar (PeNSE) 2019.

Variável		Modelo Multivariado Final			
		OR*	IC95%		Valor p
			Inferior	Superior	
Sexo					
	Masculino	1,00			
	Feminino	0,59	0,52	0,68	**0,00**
Sentir-se solitário					
	Não	1,00			
	Sim	1,25	1,05	1,49	**0,01**
Tristeza					
	Não	1,00			
	Sim	1,24	1,04	1,48	**0,02**
Tabaco regular					
	Não	1,00			
	Sim	2,21	1,87	2,63	**0,00**
Bebidas alcóolicas					
	Não	1,00			
	Sim	3,94	3,29	4,73	**0,00**
Drogas regularmente					
	Não	1,00			
	Sim	2,04	1,67	2,50	**0,00**
Amigos fumaram na sua presença					
	Não	1,00			
	Sim	2,53	2,09	3,07	**0,00**
Pais ou responsáveis fumantes					
	Não	1,00			
	Sim	1,17	1,01	1,36	**0,04**

Na análise multivariada, permaneceram associados e com maior chance de usar CE: sentir-se solitário (OR=1,25 IC95% 1,05–1,49); ter sentimentos de tristeza (OR=1,24 IC95% 1,04–1,48); fazer uso regular do tabaco (OR=2,22 IC95% 1,87–2,63); fazer uso de bebidas alcóolicas (OR=3,94; IC95% 3,29–4,73%); fazer uso de outras drogas (OR=2,04; IC95% 1,67–2,50%); ter amigos que fumaram na sua presença (OR=2,53; IC95% 2,08–3,07%) e cujos pais ou responsáveis eram fumantes (OR=1,17; IC95% 1,01–1,36%) (Tabela 2).

## DISCUSSÃO

Este estudo mediu a associação de diversas variáveis ao consumo de cigarros eletrônicos entre escolares adolescentes no Brasil em 2019. Maiores chances de utilização associaram-se aos que referiram sentir-se solitários; com sentimentos de tristeza, que faziam uso regular do tabaco, álcool e outras drogas; que tinham amigos que fumaram na sua presença; e cujos pais ou responsáveis eram fumantes.

Este é o primeiro estudo brasileiro a relatar o poliuso/ uso concomitante de diversas formas de tabaco e DEFs. O poliuso de tabaco é definido como o uso simultâneo de dois ou mais produtos de tabaco^22-36^. Entre as diversas formas de poliuso, o uso dual (cigarros convencionais e eletrônicos) foi aquele de menor proporção, com 8,56%; já a utilização combinada de todos os produtos fumados foi a maior prevalência, de 14,83%.

A publicação da PeNSE havia mostrado prevalência de uso atual de cigarros convencionais de 6,8%^20^, sendo apresentado neste estudo que o uso de outros produtos de tabaco nos 30 dias anteriores à PeNSE 2019 foi de 12,39%. Em uma revisão sistemática com metanálise de autores brasileiros, Barufaldi et al.^22^ identificaram 22 estudos longitudinais, publicados entre 2016 e 2020, de diferentes países, totalizando 97.659 participantes para o desfecho de experimentação. O uso de CE aumentou em quase três vezes e meia o risco de experimentação de cigarro comum (ou convencional) (risco relatibvo — RR=3,42; IC95% 2,81–4,15) e em mais de quatro vezes o risco de tabagismo atual (RR=4,32; IC95% 3,13–5,94)^22^.

Entre as análises de fatores associados ao uso de CE, as maiores chances de uso foi dos que referiram sentir-se solitários e com sentimentos de tristeza. O uso do tabaco tem uma relação bidirecional bem estabelecida com a saúde mental, de modo que os sintomas de saúde mental predizem o tabagismo posterior, e o tabagismo leva à deterioração dos sintomas de saúde mental^23,24^. VanFrank et al.^25^ analisaram dados autorrelatados do *National Youth Tobacco Survey* de 2024 para descrever o uso de CE e sintomas de depressão e ansiedade entre estudantes americanos do ensino fundamental (sexto a oitavo anos) e médio (anos 9–12). Entre os alunos que já haviam utilizado um produto de tabaco, 42,9% relataram uso atual. Já entre os alunos que já usaram CE, 43,6% relataram o uso atual de DEF. Em 2024, 42,1% dos jovens que atualmente usavam cigarros eletrônicos relataram sintomas moderados a graves de depressão e ansiedade, em comparação com 21,0% dos jovens que não os usavam^25^. Entre os jovens que usavam cigarros eletrônicos, aqueles com sintomas moderados a graves de depressão e ansiedade (*vs*. sintomas leves) relataram com mais frequência sintomas de dependência de nicotina^25^. Este estudo sugere que, à semelhança do que já foi estabelecido para os cigarros convencionais, pode haver uma relação bidirecional entre o uso de CE e doença mental. Em virtude do desenho de natureza transversal, não está claro se o uso de cigarros eletrônicos precedeu a piora dos sintomas de saúde mental ou se a piora dos sintomas de saúde mental precedeu o uso de cigarros eletrônicos, ou se haveria efeito em ambas as direções. Isso só pode ser avaliado por estudos de coorte longitudinais, em que a temporalidade esteja claramente estabelecida, portanto as associações aqui encontradas devem ser vistas com reserva, em função do desenho transversal do estudo.

As maiores chances de usar CE entre os adolescentes que tinham amigos que fumaram na sua presença e cujos pais ou responsáveis eram fumantes constitui um achado que está em concordância com a literatura. Le^26^ também destacou a família e os amigos como fator importante na iniciação em DEF entre adolescentes. Bailey et al.^27^ afirmaram que o uso de DEF pelos pais prediz o uso de cigarros eletrônicos pelos filhos adolescentes e adultos jovens, acima e além do uso de cigarros pelos pais. Segundo Groom et al.^28^, a influência dos colegas/amigos constitui o principal influenciador social e reforçador do uso de DEF.

A promoção e publicidade de cigarros eletrônicos e outros produtos de tabaco e nicotina é um dos principais motivadores do uso desses produtos^29^. A influência dos pares foi estudada por Kim et al.^30^: o *marketing* do estilo de vida dos CE com jovens de mesma idade ou seus colegas é característica marcante da publicidade de DEF para jovens adultos, por meio das redes sociais. A exposição à propaganda de CE foi associada com a experimentação de cigarros eletrônicos em adolescentes e adultos jovens^31^. Abreu et al.^32^ Publicaram, recentemente, a primeira investigação sobre a publicidade de produtos de tabaco e nicotina em plataformas de mídia social no Brasil. A prevalência de conteúdo voltado para públicos mais jovens alinha-se com os achados de estudos internacionais. A PeNSE 2019, como em edições anteriores, não colheu informações a respeito da exposição dos adolescentes brasileiros à promoção e publicidade de tabaco/nicotina em redes sociais.

Esta investigação revelou maior uso de CE nos adolescentes usuários de DEF que consumiam álcool. A análise de indicadores comparáveis dos escolares do 9º ano do ensino fundamental da PeNSE, municípios das capitais, no período 2009–2019, mostrou que, apesar da proibição vigente do consumo de álcool por adolescentes, 67,9% dos estudantes experimentaram bebidas alcoólicas e 25,5% consumiram-nas nos 30 dias anteriores à pesquisa^33^. A literatura mostra que o início precoce do uso de CE (definido como uso no nono ano ou anterior) foi significativamente associado ao aumento das chances de consumo de cigarros e outros comportamentos de uso de substâncias^22,34^. O uso de cigarros eletrônicos geralmente é precedido pelo uso de álcool, cigarro e maconha^34^. De fato, Milicic e Leatherdale^35^ mostraram que os jovens que bebem em excesso ou usam maconha têm maior risco de usar cigarros eletrônicos em comparação com fumantes de cigarros. Audrain-McGovern et al.^36^ mostraram que o uso de CE e narguilé à idade de 14 anos está associado com um aumento de 3,6 a 4 vezes na OR de iniciar e fazer uso atual de maconha dois anos depois. Além disso, os autores mostraram que o uso de CE e narguilé no início da adolescência mais do que dobra a OR de uso atual dual de tabaco e maconha no meio da adolescência.

Bertoni e Szklo^37^ reportaram dados do inquérito Vigilância de Fatores de Risco e Proteção para Doenças Crônicas por Inquérito Telefônico (Vigitel) de 2019, que entrevistou 52.443 indivíduos de 18 anos ou mais das 26 capitais brasileiras e do Distrito Federal. Houve maior prevalência de uso atual de DEF entre os indivíduos que fazem uso de álcool de forma abusiva do que entre os que não bebiam dessa forma (6,33 *vs*. 1,40%)^34^. Essa diferença foi mais pronunciada na faixa de 18 a 24 anos, extrato etário imediatamente superior ao avaliado pela PeNSE^37^.

Estes dados apontam claramente para a necessidade de programas de prevenção, os quais não devem ficar limitados apenas ao domínio do controle do tabaco/nicotina. Os modelos teóricos dos programas de prevenção do tabagismo em escolas são bastante variados: existem currículos de informação, influências sociais e competências sociais combinadas e multimodais^38^. O programa *Education Against Tobacco* (EAT) é um programa cuja efetividade no Brasil já foi demonstrada^39,40^.

Entretanto, faltam políticas públicas efetivas de prevenção ao uso de drogas e de sua articulação com o controle do tabaco/nicotina. Senão, vejamos: a Resolução do Conselho Nacional de Políticas sobre Drogas (CONAD) nº 08 constitui a aprovação do Plano Nacional de Políticas sobre Drogas 2022–2027^41^. O Plano basicamente se restringe à "*criação de uma plataforma para a identificação e posterior avaliação das diversas iniciativas realizadas no Brasil sobre prevenção*"^41^. Ou seja, a única diretriz até 2027 é catalogar as ações para sua posterior avaliação.

A publicação oficial da PeNSE 2019 pesquisou a forma de obtenção de produtos de tabaco dos entrevistados que já haviam experimentado cigarros: 37,5% dos experimentadores adquiriram-nos em estabelecimentos comerciais, como lojas, bares, padarias ou bancas de jornais^20^. Cabral et al.^42^ publicaram uma revisão de escopo e um relatório técnico demonstrando a importância de restringir os pontos de venda como uma das medidas para prevenir o tabagismo^43^. Ressaltam que, além de as vendas a menores de idade serem ilegais, ocorrem vendas de cigarros avulsos (unidade em vez de maço). Destacam, também, que a multiplicidade de pontos de venda (PV) facilita o acesso aos produtos de tabaco, bem como a sua existência próximo a unidades de ensino^42,43^. Os bairros de baixa renda geralmente têm uma densidade maior de PV de tabaco e apresentam maiores prevalências de seu uso, levando a iniquidades em saúde^42,43^.

Tramita no Senado Federal projeto de lei proibindo a venda de "produtos fumígenos" em estabelecimentos de ensino de níveis básico e médio, serviços de saúde, locais de venda ou consumo de alimento, supermercados, lojas de conveniência e bancas de jornais^44^. Se aprovado, constituirá um grande avanço nas políticas de controle do tabaco/nicotina em nosso país. A maior fiscalização da proibição de comercialização de DEF no país também é necessária. Perez et al. identificaram ampla comercialização ilegal de DEF e outros produtos de tabaco em *sites*, redes sociais, plataformas de *e-commerce*, desafiando as restrições impostas pela legislação brasileira^45^.

Entre os limites do estudo, destaca-se a atribuição de causalidade entre os indicadores e os desfechos investigados, em função do desenho transversal, viés de memória e possíveis erros de interpretação no questionário autoaplicado. Há viés de seleção por restringir-se a estudantes matriculados, excluindo adolescentes fora da escola, grupo potencialmente mais vulnerável. Entretanto, a PeNSE utiliza-se de uma metodologia robusta de forma a abranger todas as grandes regiões, Unidades da Federação e municípios das capitais, aproximando-se da realidade brasileira.

O estudo evidenciou a associação entre uso de CE e sofrimento mental (solidão, tristeza), sexo masculino e influência de pais ou amigos usuários. Compreender esses fatores é crucial para enfrentar a popularização da nicotina entre jovens. Destacam-se ainda o preocupante poliuso de tabaco e a forte correlação com outras substâncias: usuários de álcool e drogas ilícitas apresentaram maiores chances de utilizar cigarros eletrônicos.

Destarte, os esforços para evitar o uso de CE não devem ficar limitados ao domínio do controle do tabaco/nicotina. É urgente a articulação das políticas de controle do tabagismo e de álcool e drogas, com orquestração de ações combinadas pela Secretaria Nacional Antidrogas. É fundamental implementar ações abrangentes, incluindo aumento de preços e impostos, fiscalização da venda ilegal de cigarros avulsos e CE, restrição dos locais de venda de produtos de tabaco, campanhas de mídia (incluindo mídias sociais) e disponibilização de tratamento medicamentoso do tabagismo em grupos específicos para jovens no Sistema Único de Saúde, para, assim, lograr a redução da iniciação e da prevalência do uso de tabaco/nicotina pelos jovens brasileiros.
